# Acute kidney injury after a stroke: A PRISMA‐compliant meta‐analysis

**DOI:** 10.1002/brb3.1722

**Published:** 2020-08-05

**Authors:** Yong Huang, Chanjun Wan, Guoqing Wu

**Affiliations:** ^1^ Department of Nephrology Affiliated Hospital of Jiangxi University of Traditional Chinese Medicine Nanchang China; ^2^ Department of Cardiology Affiliated Hospital of Jiangxi University of Traditional Chinese Medicine Nanchang China

**Keywords:** acute kidney injury, meta‐analysis, stroke

## Abstract

**Background:**

The relationship between acute kidney injury (AKI) and stroke needs quantitative summary. Therefore, we analyzed the associations between AKI and stroke including the incidence, risk factors of AKI after stroke, and the influence of AKI after a stroke on prognosis of stroke.

**Methods:**

Articles published until November 2019 were searched based on the following databases: PubMed, Web of Science, EMBASE, Medline, and Google Scholar. We computed the following results [rates of AKI incidence after a stroke, odds ratios (ORs) or relative risks (RRs) estimates and the 95% confidence intervals (CIs) for the association between risk factors and AKI, ORs or RRs and the CIs for the association between AKI and outcomes after a stroke] by using STATA 13.0 software.

**Results:**

The study reported an overall incidence of AKI of 12% with a random‐effects model. Additionally, the present study showed that higher National Institutes of Health Stroke Scale (NIHSS) score on admission and history of hypertension were associated with higher risk of AKI after stroke. Moreover, the study showed that AKI after stroke was associated with higher in‐hospital mortality, higher 1‐month mortality, higher long‐term mortality, and poorer functional outcome.

**Conclusions:**

Acute kidney injury appears to be a common complication after stroke and is related to increased mortality and disability in stroke. Additionally, high NIHSS score on admission and history of hypertension were the critical risk factors for the AKI after stroke. More large‐scale studies should be made to explore AKI after stroke.

## Graphical abstract

AKI appears to be a common complication after stroke and is related to increased mortality and disability in stroke. High NIHSS score on admission and history of hypertension were the critical risk factors for the AKI after stroke.

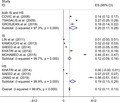

## 1 INTRODUCTION

Stroke is a severe nervous system disease and can easily lead to the death or disability of patients. The limited blood flow to brain is the main cause of stroke. According to the type of hypoperfusion, stroke is classified as ischemic stroke (IS) or hemorrhagic stroke (HS) (Koh & Park, 2017). 80% of all strokes are ischemic stroke; hemorrhagic stroke accounts for about 20% (Boursin, Paternotte, Dercy, Sabben, & Maïer, 2018). According to previous reports, stroke is the second deadliest disease and a major disease of high crippling rate worldwide (Katan & Luft, 2018). The use of several preventive drugs, including hypotensive and antihypercholesterolemic drugs, may be the main reason of decreased incidence of new and recurrent stroke (Guzik & Bushnell, 2017).

Acute kidney injury (AKI) is extremely common in hospitalized patients with poor prognosis, especially in intensive care unit (ICU) patients, and characterized by a sudden decrease of the kidney's excretory function (Koza, 2016). The incidence of AKI in stroke patients ranges from 8% to 21% (Khatri et al., 2014). Additionally, the physiological changes including blood pressure, hormone levels, physical disability, and the treatments of stroke may promote the occurrence and development of AKI after stroke (Arnold et al., 2018). Moreover, several studies have proved that the occurrence of AKI after stroke is associated with the increased mortality (Khatri et al., 2014; Snarska, Kapica‐Topczewska, Bachórzewska‐Gajewska, & Małyszko, 2016). The relationship between AKI and stroke needs quantitative summary. Therefore, we analyzed the associations between AKI and stroke including the incidence, risk factors of AKI after stroke, and the influence of AKI after a stroke on prognosis of stroke.

## 2 METHODS

The meta‐analysis was performed based on the Preferred Reporting Items for Systematic reviews and Meta‐Analysis (PRISMA) guideline (Moher, Liberati, Tetzlaff, & Altman, 2009).

### 2.1 Search strategy and selection criteria

The following databases (PubMed, Web of Science, EMBASE, Medline, and Google Scholar) were selected to search for articles published in English before November 2019. Search terms included (“acute kidney injury” OR “acute kidney failure” OR “acute renal failure”) AND (“stroke”). The study included studies reporting rates of AKI incidence after a stroke, risk factors for AKI after stroke, or the associations between AKI and outcomes after a stroke. Studies that did not explore the AKI after a stroke were excluded from the study. Moreover, we excluded reviews, case reports, and meta‐analyses.

### 2.2 Data extraction

After reading the full text, we extracted data as follows: author, publication year, study type, study location, sample size, information of included participants (age and gender), diagnosis method of AKI, chronic kidney disease (CKD) excluded, follow‐up time, length of stay in hospital, and results [rates of AKI incidence after a stroke, odds ratios (ORs) or relative risks (RRs) estimates and the 95% confidence intervals (CIs) for the association between risk factors and AKI, ORs or RRs and the CIs for the association between AKI and outcomes after a stroke].

### 2.3 Meta‐analysis

*Q* test and *I* ^2^ were used to explore heterogeneities between studies by using STATA 13.0 software. Random‐effects models were performed as pooling methods with invariably high heterogeneity (*p* value for *Q* test ≤ 0.05 and *I* ^2^ ≥ 50%); otherwise, fixed‐effects models were conducted as pooling methods with invariably low heterogeneity (*p* value for *Q* test > 0.05). Subgroup analyses (for types of stroke) were conducted to explore source of the heterogeneity. We assessed publication bias with Begg's test, Egger's test, and funnel plot.

## 3 RESULTS

### 3.1 Study selection and characteristics

**FIGURE 1:**
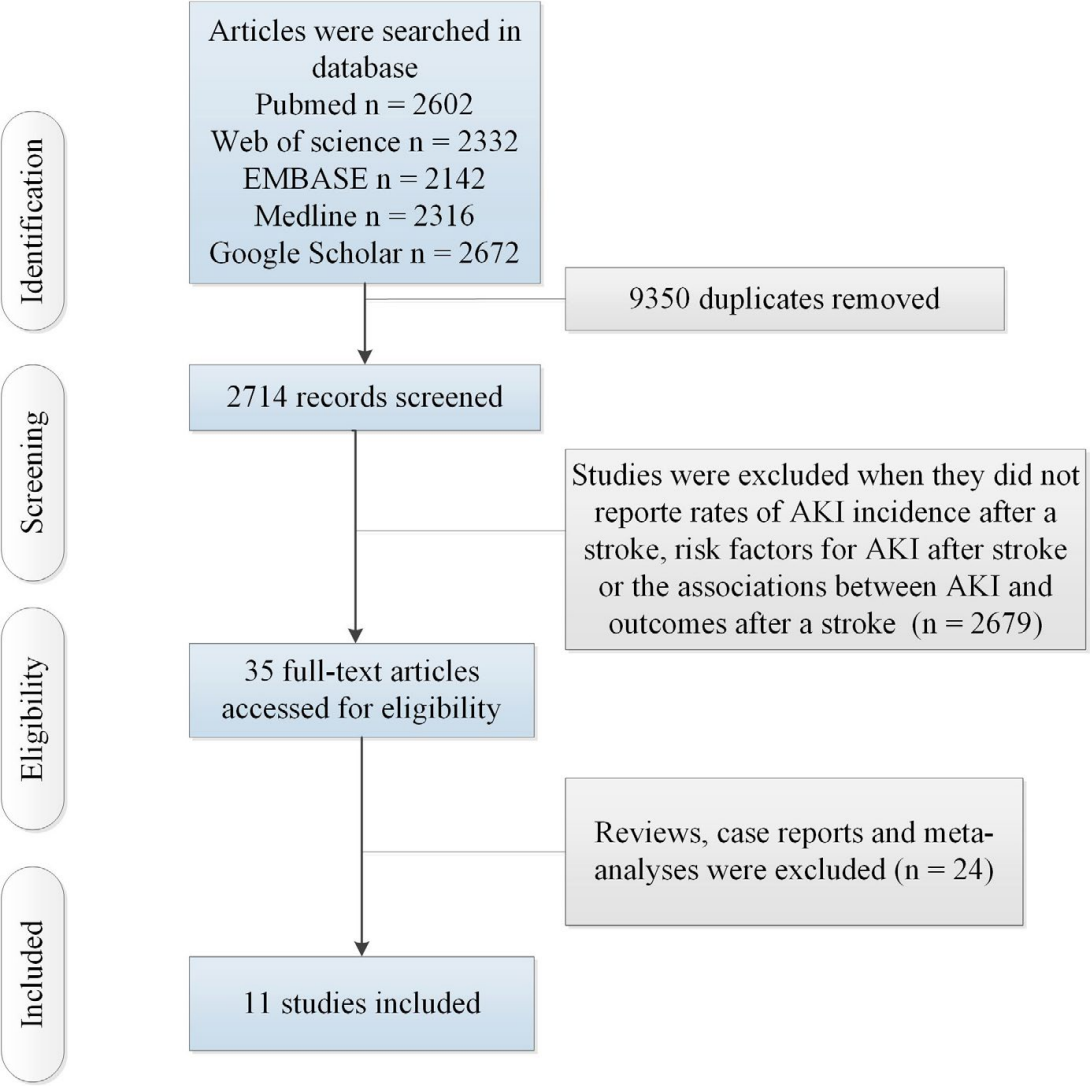
Flow of information through the different phases of a meta‐analysis

Figure 1 shows the selection process and finally included studies. Table S1 showed the characteristics of 11 finally included studies. The study included two prospective observational studies [including 4,515 ischemic stroke (IS) and 323 hemorrhagic stroke (HS) patients] (Lin et al., 2011; Tsagalis et al., 2009) and nine retrospective observational studies (including 11,014,783 IS and 1,312,531 HS patients) (Covic et al., 2008; Grosjean et al., 2019; Jiang et al., 2019; Kamouchi et al., 2013; Khatri et al., 2014; Mohamed, Bhattacharya, Shankar, Chaturvedi, & Madhavan, 2015; Nadkarni et al., 2015; Saeed et al., 2014; Saeed, Adil, Piracha, & Qureshi, 2015).

### 3.2 Results of meta‐analysis

**FIGURE 2:**
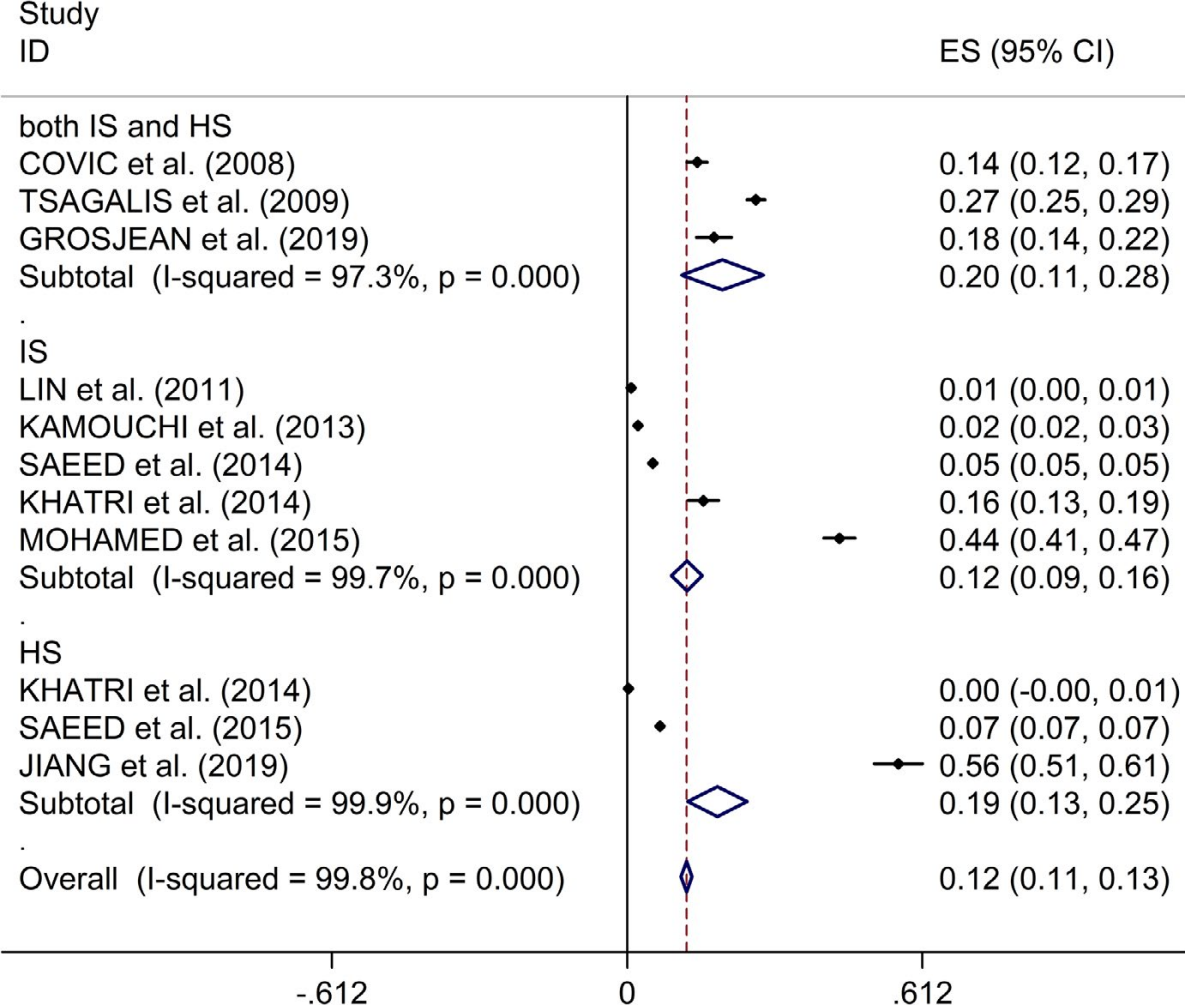
Forest plots of incidence of AKI after stroke. AKI, acute kidney injury

The study reported an overall incidence of AKI of 12% with a random‐effects model (incidence 12%, 95% CI 11%‐13%, *I* ^2^ = 99.8%,* p* < .001, Figure 2). Additionally, the incidence was 12% with IS and 19% in HS (IS: incidence 12%, 95% CI 9%‐16%, *I* ^2^ = 99.7%,* p* < .001; HS: incidence 19%, 95% CI 13%‐25%, *I* ^2^ = 99.9%,* p* < .001, Figure 2). Begg's test, Egger's tests, and funnel plots showed no significant publication bias between included studies (Begg's test: *p* = .161; Egger's test: *p* = .480; Figure S1).

**FIGURE 3:**
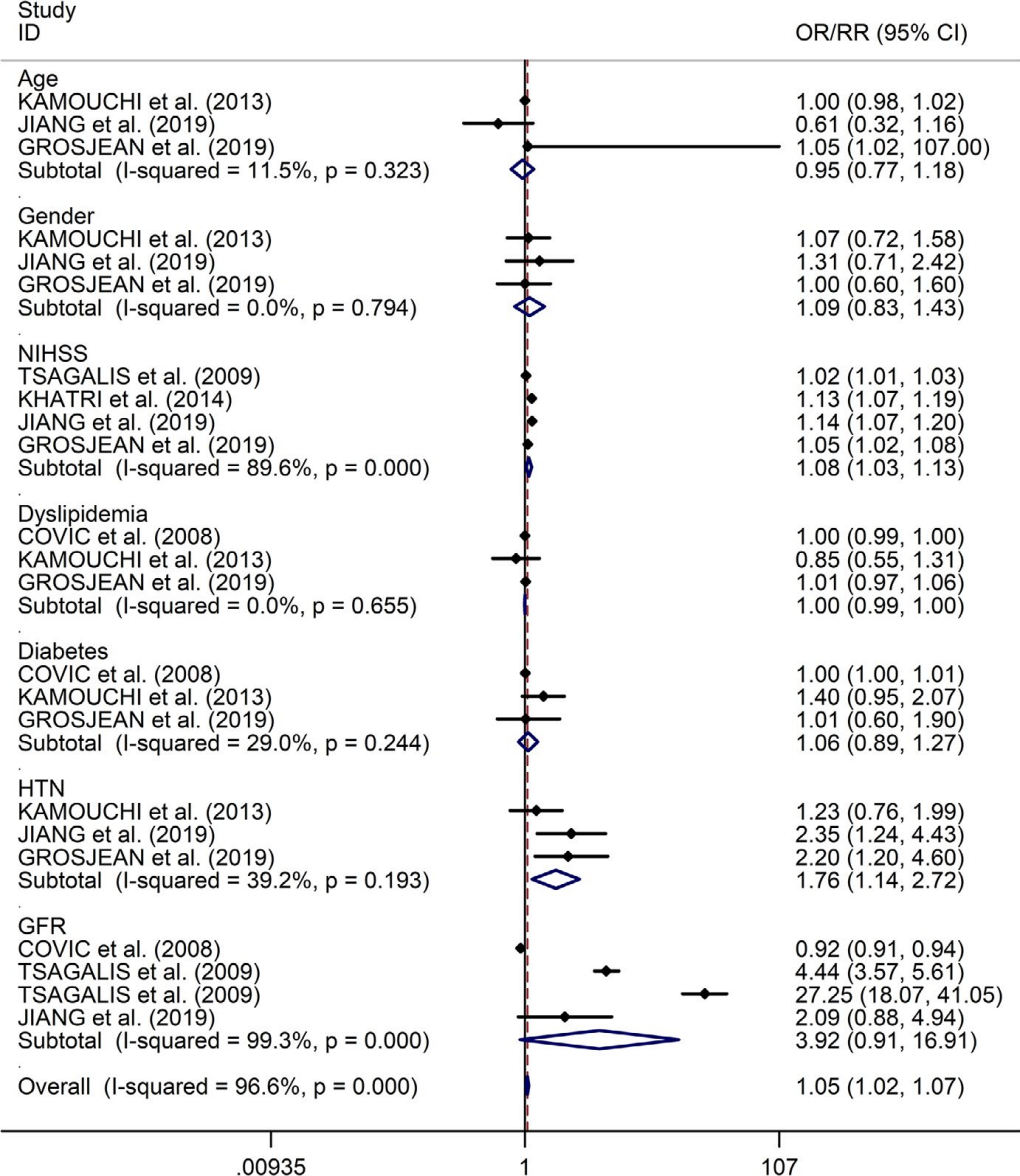
Forest plots of associations between various risk factors and risk of AKI after stroke. AKI, acute kidney injury

The present study showed that higher National Institutes of Health Stroke Scale (NIHSS) score on admission and history of hypertension were associated with higher risk of AKI after stroke (NIHSS: OR/RR 1.08, 95% CI 1.03–1.13, *I* ^2^ = 89.6%,* p* < .001; history of hypertension: OR/RR 1.76, 95% CI 1.14–2.72, *I* ^2^ = 39.2%,* p* = .193, Figure 3), whereas no significant associations were detected between risk of AKI after stroke and age, gender, dyslipidemia, history of diabetes, and baseline glomerular filtration rate (GFR) on admission (age: OR/RR 0.95, 95% CI 0.77–1.18, *I* ^2^ = 11.5%,* p* = .323; gender: OR/RR 1.09, 95% CI 0.83–1.43, *I* ^2^ = 0.0%,* p* = .794; dyslipidemia: OR/RR 1.00, 95% CI 0.99–1.00, *I* ^2^ = 0.0%,* p* = .655; history of diabetes: OR/RR 1.06, 95% CI 0.89–1.27, *I* ^2^ = 29.0%,* p* = .244; GFR on admission: OR/RR 3.92, 95% CI 0.91–16.91, *I* ^2^ = 99.3%,* p* < .001, Figure 3).

**FIGURE 4:**
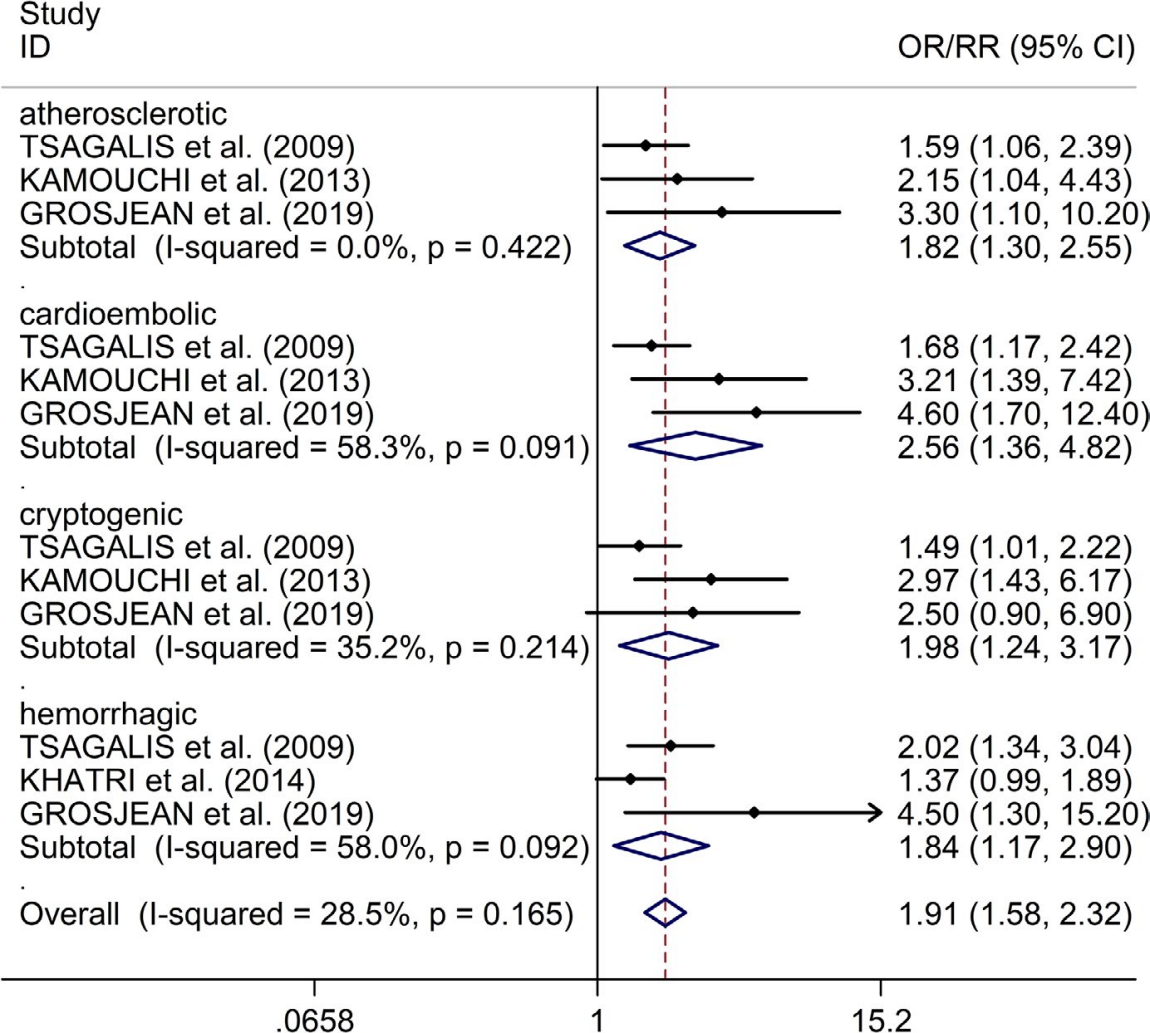
Forest plots of associations between different types of stroke and risk of AKI after stroke. AKI, acute kidney injury

The study explored the association between types of stroke and risk of AKI after stroke. The study showed that with respect to lacunar stroke, another four types of stroke (atherothrombotic IS, cardioembolic IS, unclassified IS, and HS) were associated with higher risk of AKI after stroke (atherothrombotic IS: OR/RR 1.82, 95% CI 1.30–2.55, *I* ^2^ = 0.0%,* p* = .422; cardioembolic IS: OR/RR 2.56, 95% CI 1.36–4.82, *I* ^2^ = 58.3%,* p* = .091; unclassified IS: OR/RR 1.98, 95% CI 1.24–3.17, *I* ^2^ = 35.2%,* p* = .214; HS: OR/RR 1.84, 95% CI 1.17–2.90, *I* ^2^ = 58.0%,* p* = .092, Figure 4).

**FIGURE 5:**
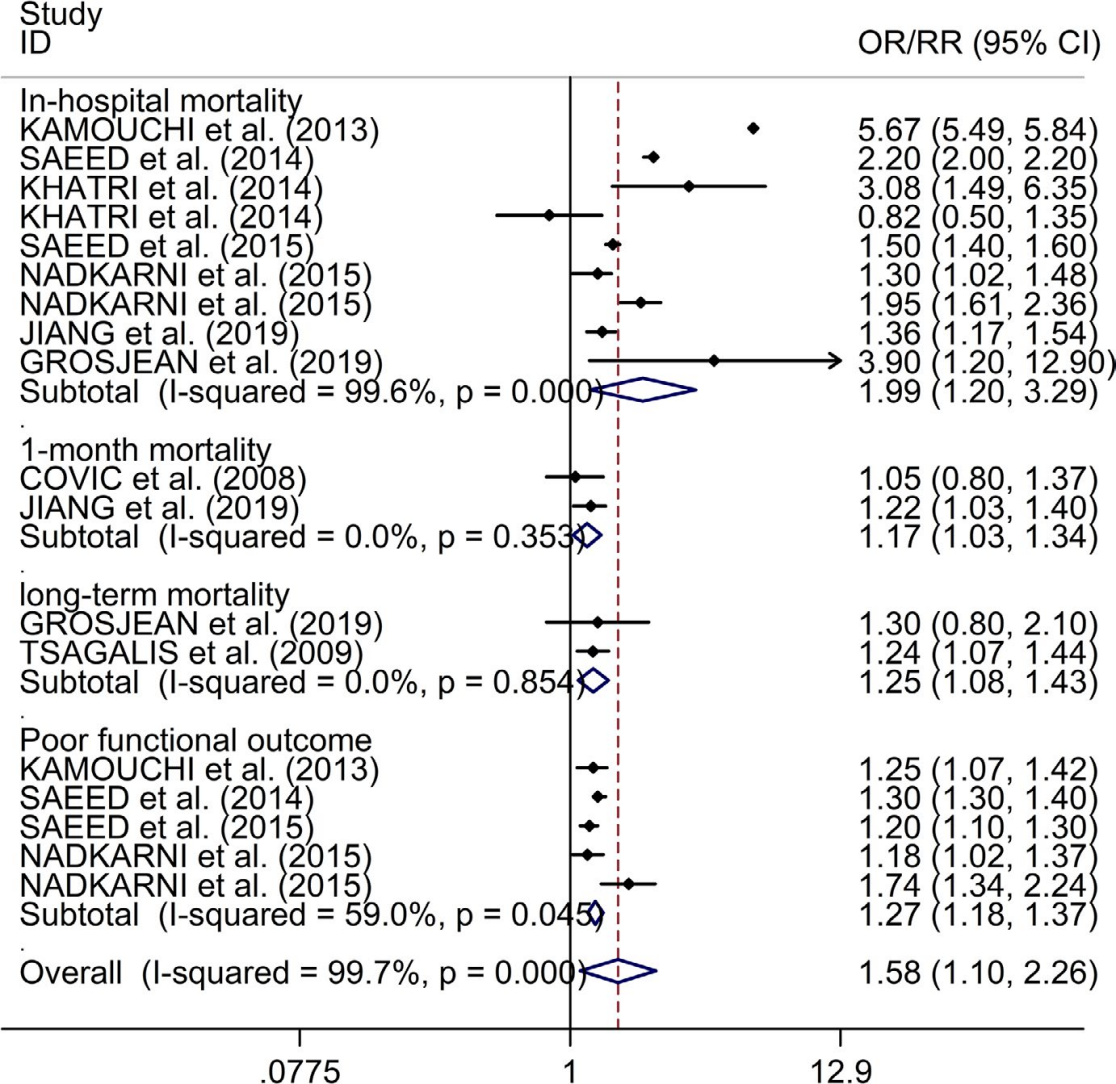
Forest plots of associations between AKI after stroke and in‐hospital mortality, 1‐month mortality, long‐term mortality, and functional outcome. AKI, acute kidney injury

**FIGURE 6:**
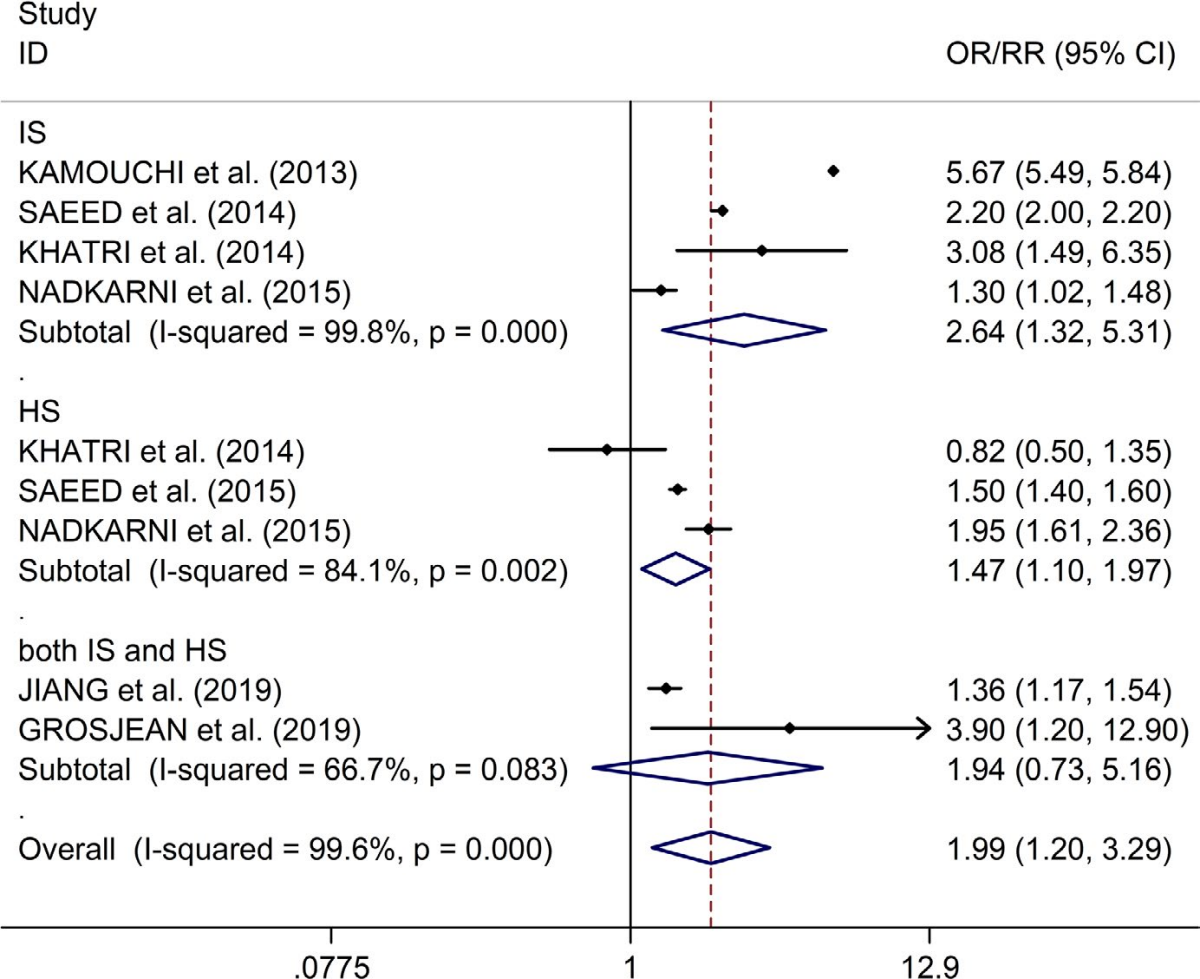
Subgroup analysis regarding associations between AKI after stroke and in‐hospital mortality in IS and HS. AKI, acute kidney injury; HS, hemorrhagic stroke; IS, ischemic stroke

**FIGURE 7:**
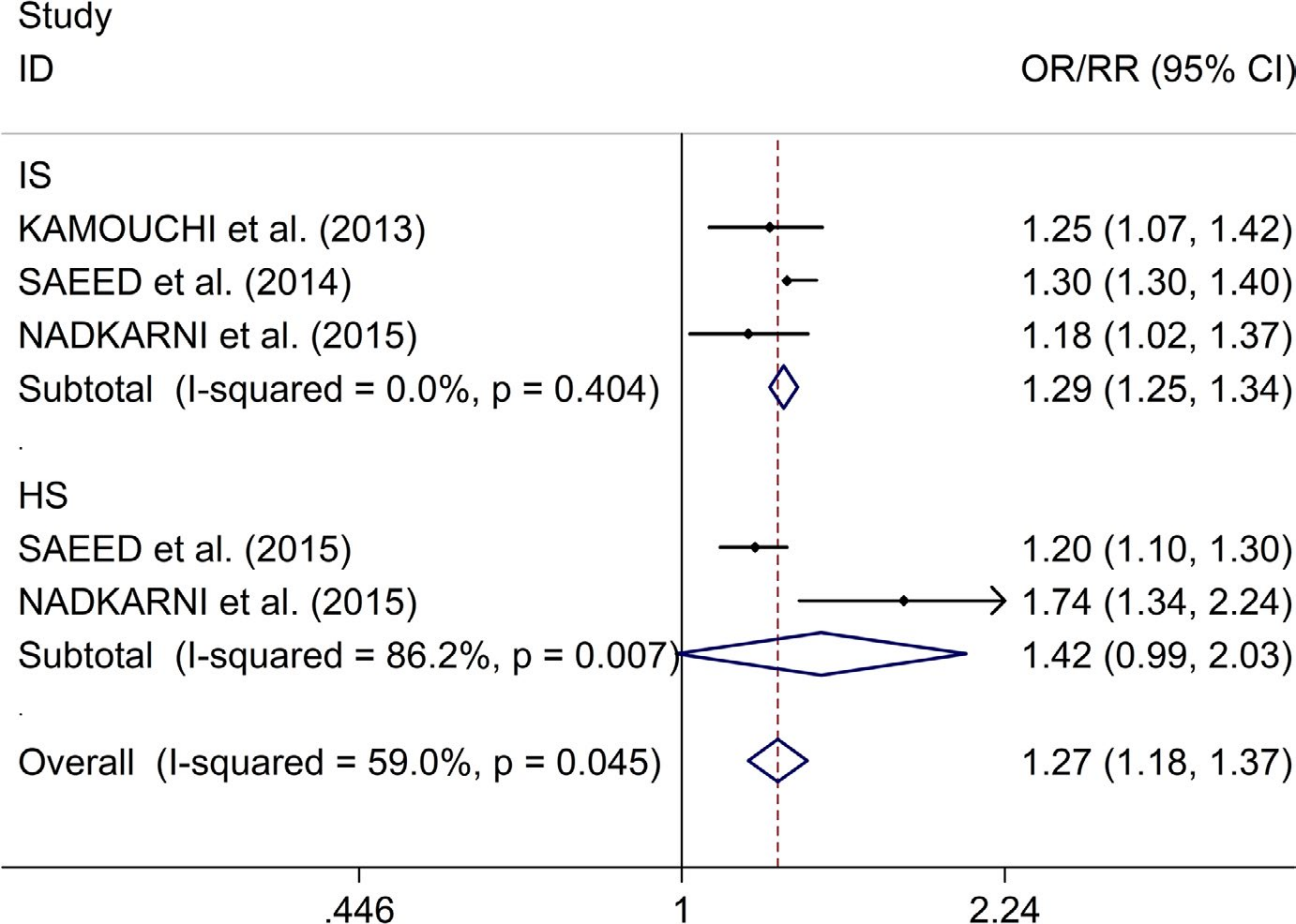
Subgroup analysis regarding associations between AKI after stroke and functional outcome in IS and HS. AKI, acute kidney injury; HS, hemorrhagic stroke; IS, ischemic stroke

Additionally, the study showed that AKI after stroke was associated with higher in‐hospital mortality, higher 1‐month mortality, higher long‐term mortality, and poorer functional outcome (in‐hospital mortality: OR/RR 1.99, 95% CI 1. 20–3.29, *I* ^2^ = 99.6%,* p* < .001; 1‐month mortality: OR/RR 1.17, 95% CI 1.03–1.34, *I* ^2^ = 0.0%,* p* = .353; long‐term mortality: OR/RR 1.25, 95% CI 1.08–1.43, *I* ^2^ = 0.0%,* p* = .854; poorer functional outcome: OR/RR 1.27, 95% CI 1.18–1.37, *I* ^2^ = 59.0%,* p* = .045, Figure 5). Regarding the association between AKI after stroke and in‐hospital mortality, subgroup study showed that AKI after stroke was associated with higher in‐hospital mortality in both IS and HS (IS: OR/RR 2.64, 95% CI 1.32–5.31, *I* ^2^ = 99.8%,* p* < .001; HS: OR/RR 1.47, 95% CI 1.10–1.97, *I* ^2^ = 84.1%,* p* = .002, Figure 6). Additionally, Begg's test, Egger's tests, and funnel plots showed no significant publication bias between included studies (Begg's test: *p* = .076; Egger's test: *p* = .176; Figure S2). Regarding the association between AKI after stroke and poor functional outcome, subgroup study showed that AKI after stroke was associated with poorer functional outcome in IS (OR/RR 1.29, 95% CI 1.25–1.34, *I* ^2^ = 0.0%,* p* = .404, Figure 7), whereas no significant association was indicated between AKI after stroke and poor functional outcome in HS (OR/RR 1.42, 95% CI 0.99–2.03, *I* ^2^ = 86.2%,* p* = .007, Figure 7). Additionally, Begg's test, Egger's tests, and funnel plots showed no significant publication bias between included studies (Begg's test: *p* = .806; Egger's test: *p* = .998; Figure S3).

## 4 DISCUSSION

In our study, we reported the rate of AKI after stroke was 12% (95% CI 11%‐13%), indicating a significant relationship between stroke and AKI after stroke. And for HS patients, the incidence rate of AKI after stroke was higher (HS: incidence 19%, 95% CI 13%‐25%; IS: incidence 12%, 95% CI 9%‐16%). Our result was consistent with previous results (Khatri et al., 2014; Zorrilla‐Vaca et al., 2018). However, the reported rates of AKI secondary to stroke ranged from 0.82% to 26.68% (Arnold et al., (2018)). The differences in the diagnostic criteria of AKI may be the major reasons for the various reported rates. We noticed that the diagnosis of AKI was based on different standards including end‐stage kidney disease (ESKD) classification, AKI network criteria, and kidney disease: improving global outcomes (KDIGO) criterion (Jiang et al., 2019; Mehta et al., 2007). Thus, a unified criterion for AKI should be applied during the evaluation process regarding the incidence of AKI after stroke.

The present study demonstrated that high NIHSS score on admission and history of hypertension were the risk factors for the AKI patients secondary to stroke, whereas several factors (such as age, gender, lipid abnormality, history of diabetes, and baseline GFR on admission) did not affect the occurrence of AKI following stroke. The NIHSS is used to evaluate the severity and prognosis of stroke (Adams et al., 1999). Wang et al. (2018) found that the incidence of AKI in the NICU patients was 20.9% and suggested that the patients with high NIHSS score were easily to suffer from AKI because of severe illness and infectious complications. Hypertension is a risk factor for stroke because of the vascular changes caused by blood pressure change (Cipolla, Liebeskind, & Chan, 2018). Lapi, Azoulay, Yin, Nessim, and Suissa (2013) reported that combined use of several antihypertensive drugs was associated with an increased risk of AKI. Of note, more researches are needed to explain whether the unrelated factors are risk factors or not such as diabetes, baseline GFR, and dyslipidemia.

Among IS subtypes, atherothrombotic IS, cardioembolic IS, and unclassified IS were associated with higher AKI risk than lacunar IS. The result was consistent with previous studies (Grosjean et al., 2019). Compared with other type of IS, lacunar IS is less neurologically severe (Makin, Turpin, Dennis, & Wardlaw, 2013). Maybe this is why other types of stroke are more associated with AKI secondary to stroke than lacunar IS.

In our study, we found that the patients of AKI after stroke were associated with worse outcomes, including higher in‐hospital mortality, 1‐month mortality, and long‐term mortality, and poorer functional outcome. Indeed, the period of admission is very critical in AIS and the AKI could be related to the use of contrast‐enhanced image studies or other in‐hospital abnormalities (infection, disability, etc.). However, some studies included in the present study did not explore the impact of these indicators on the association between AKI after stroke and mortality. For stroke patients, the occurrence and development of AKI were disadvantageous. Both IS and HS were significant related to higher in‐hospital mortality in subgroup study. However, our study indicated that only IS was associated with poor functional outcome. However, we should notice that only two studies were included to explore the relationship between HS and poor functional outcome.

There were several limitations in the present study. First, the different diagnostic criteria of AKI used in various studies may affect the selection of sample group. The differences of sample group may influence the judgment of the association between stroke and AKI. Second, the number of included studies was small, and the heterogeneity between studies may restrict our conclusions. We need more high‐quality studies to include in our analysis. Third, most studies included in the present study had a short follow‐up, usually until the hospital discharge (only one has 10‐year follow‐up). Fourth, the present study did not explore the impact of period of admission and impact of the use of contrast‐enhanced image studies or other in‐hospital abnormalities on the association between AKI after stroke and mortality.

## 5 CONCLUSIONS

AKI appears to be a common complication after stroke and is related to increased mortality and disability in stroke. Additionally, high NIHSS score on admission and history of hypertension were the critical risk factors for the AKI after stroke. More large‐scale studies should be made to explore AKI after stroke.

## CONFLICT OF INTERESTS

No conflict of interests.

## AUTHOR CONTRIBUTION

Yong Huang analyzed the data and worked substantially on paper drafting. Chanjun Wan analyzed the data. Guoqing Wu was involved in the review and editing of the manuscripts.

## PEER REVIEW

The peer review history for this article is available at https://publons.com/publon/10.1111/brb3.1722[Correction added on September 9,2020 , after first online publication: Peer review history statement has been added.]

## ETHICAL STATEMENT

The present study was a meta‐analysis. Thus, ethical statement is not provided.

## Supporting information

Supplementary Material

## Data Availability

The study was a meta‐analysis. I have attached all data in Table S1.
