# Supplementary material for: Acute kidney injury after a stroke: A PRISMA‐compliant meta‐analysis
Source: Brain Behav. 2020 Aug 5;10(9):e01722. doi: 10.1002/brb3.1722 (PMC7507391; doi:10.1002/brb3.1722)
Supplement: Supplementary file 1 — Supplementary Material [file BRB3-10-e01722-s001.docx]

Supplementary table 1. Characteristics of all included studies.

| References | Study design | Country | Sample Size | Gender (male%) | Mean Age (SD) | AKI  definition | period of admission | the use of contrast-enhanced image or other in-hospital abnormalities | CKD  excluded? | Follow  up | LOS (days)  and AKI | Results |
| --- | --- | --- | --- | --- | --- | --- | --- | --- | --- | --- | --- | --- |
| COVIC et al. (2008) [*^1^*](#_ENREF_1) | Retrospective observational study | Romania | 1090 (IS 932, HS 158) | 49.3% | 66.1 ± 11.5 | Creatinine  values;  RIFLE | NR | NR | No | 30 days | NR | **Incidence:** 14.5%; **Factors:** GFR: 0.924 (0.911, 0.936), IHD: 0.564 (0.336, 0.947), CHF: 0.526 (0.326, 0.850), Serum triglycerides: 0.997 (0.994, 1.000), Serum glucose: 1.003 (1.000, 1.006), Type of stroke: 2.504 (1.423, 4.406); ACE inhibitors/AT-II receptor blockers: 1.004 (0.993, 1.058); **30-day mortality**: 1.05 (0.8, 1.37) |
| TSAGALIS et al. (2009) [*^2^*](#_ENREF_2) | Prospective observational study | Greece | 2155 (1832 IS, 323 HS) | 61.2% | 70.3 ± 11.9 | Creatinine  values;  AKIN | 5.7 ± 6.8 h | NR | No | 10 years | NR | **Incidence:** 26.68%; **Factors:** NIHSS: 1.02 (1.01, 1.03); HF: 1.47 (1.01, 2.13); GFR: [30 to 60: 4.44 (3.57, 5.61); ＜30 27.25 (18.07, 41.05); Stroke subtype: [atherosclerotic: 1.59 (1.06, 2.39; cardioembolic: 1.68 (1.17, 2.42); cryptogenic: 1.49 (1.01, 2.22); hemorrhagic: 2.02 (1.34, 3.04); **10-year** **mortality:** HR 1.24 (1.07, 1.44); |
| LIN et al. (2011) [*^3^*](#_ENREF_3) | Prospective observational study | China | 2683 IS | 58.4% | 66.1 ± 13.59 (AF),  63.58 ± 13.64  (no AF) | ICD-10  coding | within 1 month | NR | No | 1 year | NR | **Incidence**: 0.82% |
| KAMOUCHI et al. (2013) [*^4^*](#_ENREF_4) | Retrospective observational study | Japan | 5689 IS | Non-AKI 59.4%, AKI 58.6% | Non-AKI 72.4 ± 11.9, AKI 73.7 ± 9.95 | Creatinine  values | NR | Infection difference | No | Hospital  discharge |  | **Incidence**: 2.24%; **Factors:** Age: 1.00 (0.98-1.02); Female: 1.07 (0.72-1.58); Hypertension: 1.23 (0.76-1.99); Dyslipidemia: 0.85 (0.55-1.31); Diabetes: 1.40 (0.95-2.07); Atrial fibrillation 1.57 (0.93-2.65); Stroke subtypes: Atherothrombotic 2.15 (1.04-4.43); Cardioembolic 3.21 (1.39-7.42); Unclassifie 2.97 (1.43-6.17); NIHSS Q2 (3-4) 1.53 (0.84-2.78); NIHSS Q3 (5-10) 1.42 (0.80-2.52); NIHSS Q4 (11-42) 1.56 (0.84-2.89); SBP: 1.00 (0.99-1.01); creatinine: 1.40 (1.30-1.50); Infectious complications: 1.61 (1.04-2.49); Stroke therapy started on admission day: Free radical scavenger 0.45 (0.30-.67); **In-hospital mortality:** 5.667 (5.492- 5.842) ; **Poor functional outcome:** 1.245 (1.070 1.420). |
| SAEED et al. (2014) [*^5^*](#_ENREF_5) | Retrospective observational study | USA | 7,068,334 IS | 46.1% | No AKI 71 ± 31,  AKI 74 ± 28 | ICD-9  coding | NR | NR | Yes | Hospital  discharge | Unadjusted 6 vs 4 days (*p* < 0.0001) | **Incidence:** 5.27%; **In-hospital mortality:** 2.2 (2.0, 2.2); **moderate/severe disability**: 1.3 (1.3,1.4); |
| KHATRI et al. (2014) [*^6^*](#_ENREF_6) | Retrospective observational study | USA | 1357 (IS 528, HS 829) | 56% | 64 ± 16 | Creatinine  values;  AKIN | NR | NR | GFR  < 15 ml/min  excluded | Hospital  discharge | Unadjusted  AIS: 17.6 vs  8.4 days  (P ≤ 0.001)  ICH: 13.0 vs  8.0 days  (P ≤ 0.001) | **Incidence:** total: 17.9%, stroke: IS: 15.8%, HS: 26%; **Factors:** Baseline creatinine: 1.61 (1.22-2.12); NIHSS score: 1.13 (1.07-1.19); Smoking: 0.57 (0.38-0.85); HS: 1.37 (0.99-1.89); **In-hospital mortality:** IS: 3.08 (1.49, 6.35), HS: 0.82 (0.50, 1.35) |
| SAEED et al. (2015) [*^7^*](#_ENREF_7) | Retrospective observational study | USA | 614,454 HS | 52.2% | No AKI 69 ± 37,  AKI 68 ± 34 | ICD-9  coding | NR | In-hospital complications | Yes | Hospital  discharge | Unadjusted  12 vs 7 days  (*p* < 0.0001) | **Incidence:**6.79%; **In-hospital mortality:** 1.5 (1.4, 1.6); **moderate/severe disability**: 1.2 (1.1, 1.3) |
| NADKARNI et al. (2015) [*^8^*](#_ENREF_8) | Retrospective observational study | USA | 4,634,682 (3,937,928 IS, 696,754 HS) | IS 50.0%, HS: 60.0% | IS: No AKI 73 ± 0.2, AKI 66 ± 0.3 HS: No AKI 69.7 ± 0.13, AKI 65.4 ± 0.21 | ICD-9 coding AKI-D only | NR | NR | No | Hospital  discharge | Unadjusted IS: 14.1 vs 3.6 days (P ≤ 0.01)  HS: 23.5 vs 5.3 days (P ≤ 0.01) | **In-hospital mortality:** IS: OR 1.30 (1.02, 1.48); HS: OR 1.95 (1.61, 2.36); **adverse discharge category:** IS: 1.18 (1.02, 1.37); HS: 1.74 (1.34, 2.24) |
| MOHAMED et al. (2015) [*^9^*](#_ENREF_9) | Retrospective observational study, for AKI-D | USA | 897 IS | 44.0% | 64.4 ± 14.7 | ICD-9  coding | NR | NR | No | Hospital  discharge | 2.63 (1.51-4.58)  Adjusted for  comorbidities,  complications,  NIHSS score | **Incidence**: 7.92%; |
| JIANG et al. (2019) [*^10^*](#_ENREF_10) | Retrospective observational study | China | 381: 79 IS, 302 HS | 56.2% | 61 (51-69) | KDIGO criteria | NR | NR | No | Hospital  discharge | 18 days vs 19 days, *p* = 0.244 | **Incidence**: 30.2%; **Factors:** Hypertension 2.346 (1.244–4.426), Loop diuretics 1.961 (1.060–3.625), NIHSS 1.136 (1.074–1.202), APACHE II 1.107 (1.004–1.220), sCysC 8.156 (2.353–28.272), Age >65 years 0.613 (0.324–1.161), Gender 1.307 (0.706–2.421), sCr 0.988 (0.975–1.001), β2-MG 1.000 (1.000–1.001), eGFR 2.085 (0.880–4.941), BUN 1.009 (0.923–1.104), CKD 0.383 (0.023–6.460), Vancomycin 8.619 (0.337–220.307), Vasoactive drug 1.825 (0.988–3.370), Serum sodium 1.006 (0.959–1.055), Leucocyte 1.025 (0.979–1.074); **In-hospital mortality:** 1.356 (1.171, 1.541); **28-day mortality:** 1.216 (1.030, 1.401) |
| GROSJEAN et al. (2019) [*^11^*](#_ENREF_11) | Retrospective observational study | Italy | 430: 396 IS, 34 HS | 51% | 75 ± 12 | Creatinine  values; | NR | NR | No | 19.2 months | 12 days vs 10 days, *p* = 0.01 | **Incidence**: 18.0%; **Factors:** age 1.05 (1.02–107), gender 1.0 (0.6–1.6), DM 1.01 (0.6–1.9), HTN 2.2 (1.2–4.6), CKD 4.5 (2.4–8.2), AF 2.0 (1.2–3.3), Obesity 1.1 (0.6–2.0), Charlson CI 1.2 (1.01–1.3), SBP 0.87 (0.78–0.98), DBP 0.80 (0.68–0.99), HR 1.0 (0.99–1.01), TCHOL 0.91 (0.85–0.97), TGL 1.01 (0.97–1.06), WBC 1.03 (0.95–1.11), HTC 0.93 (0.89–0.97), TNI 0.96 (0.83–1.11), CRP 0.94 (0.93–0.96), UPCR 2.8 (1.2–6.7), NIHSS 1.05 (1.02–1.08), Stroke cause: Cardioembolism 4.6 (1.7–12.4), Large artery atherosclerosis 3.3 (1.1–10.2), Haemorrhagic 4.5 (1.3–15.2), Arterial dissection and rare cause 2.3 (0.2–22.7), Undetermined 2.5 (0.9–6.9); **In-hospital mortality:** 3.9 (1.2–12.9); **long-term mortality:** 1.3 (0.8–2.1) |

Abbreviations: ACE, angiotensin-converting enzyme; AF, history of atrial fibrillation; AKI-D, acute kidney injury-dialysis; Charlson CI, Charlson Comorbidity Index; CHF, chronic heart failure; CKD, chronic kidney disease; CRP, c-reactive protein; DBP, diastolic blood pressure; DM, diabetes mellitus; eGFR, estimated glomerular filtration rate; GFR, glomerular filtration rate; HR, heart rate; HS, haemorrhagic stroke; HTC, haematocrit; IHD, ischaemic heart disease; HTN, history of systemic hypertension; IQR, inter quantile range; IS, ischaemic stroke; KDIGO, Kidney Disease Improving Global Outcomes; LOS, length of stay; NIHSS, NIH Stroke Scale/Score; NR, not reported; SBP, systolic blood pressure;

sCysC, serum cystatin C; TCHOL, total cholesterol; TGL, triglycerides; TNI, troponin I; UPCR, urinary protein to creatinine ratio; WBC, white blood cells count;β2-MG, β2-microglobulin.


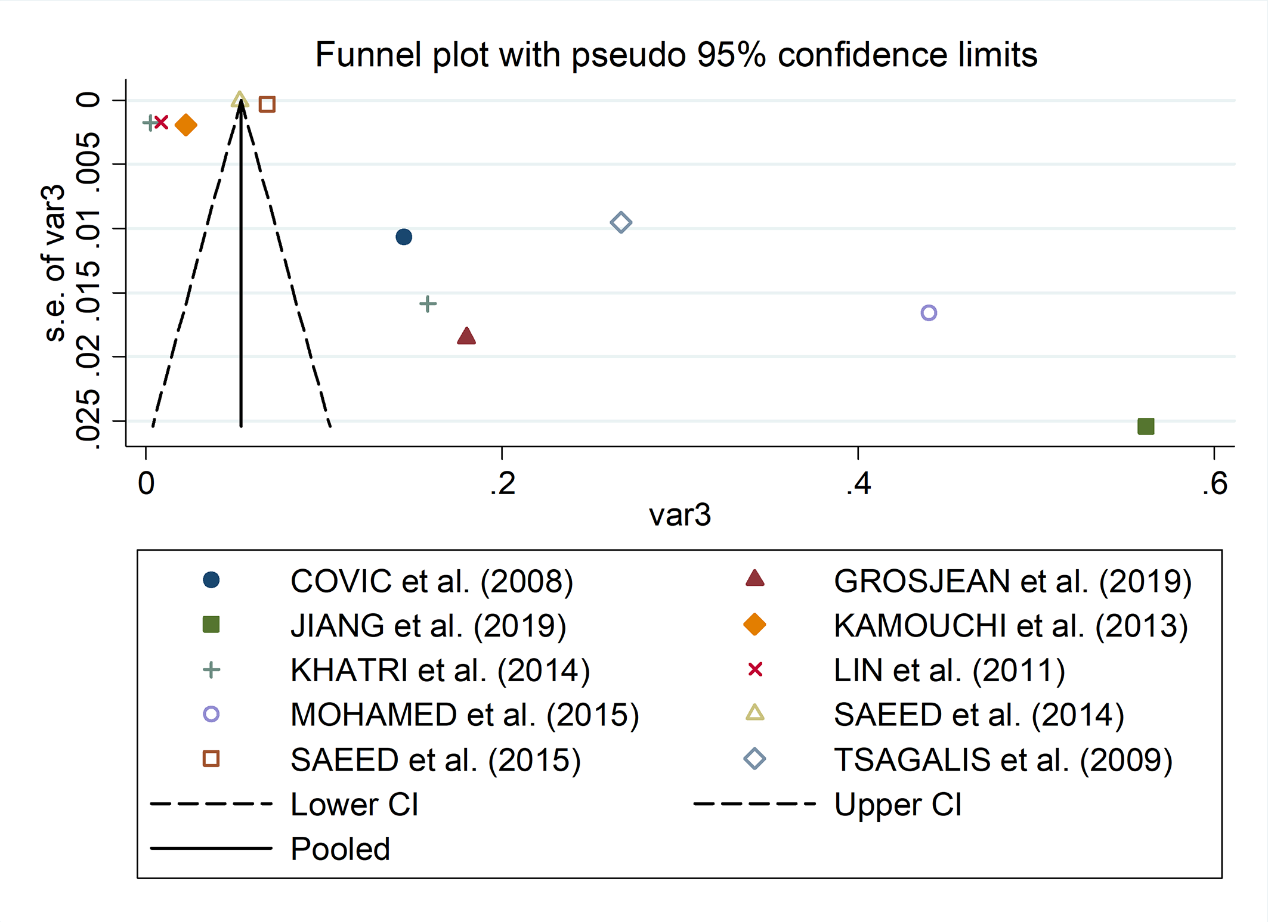


Supplementary figure 1. Funnel plots of studies exploring incidence of AKI after stroke. Abbreviation: AKI, acute kidney injury.


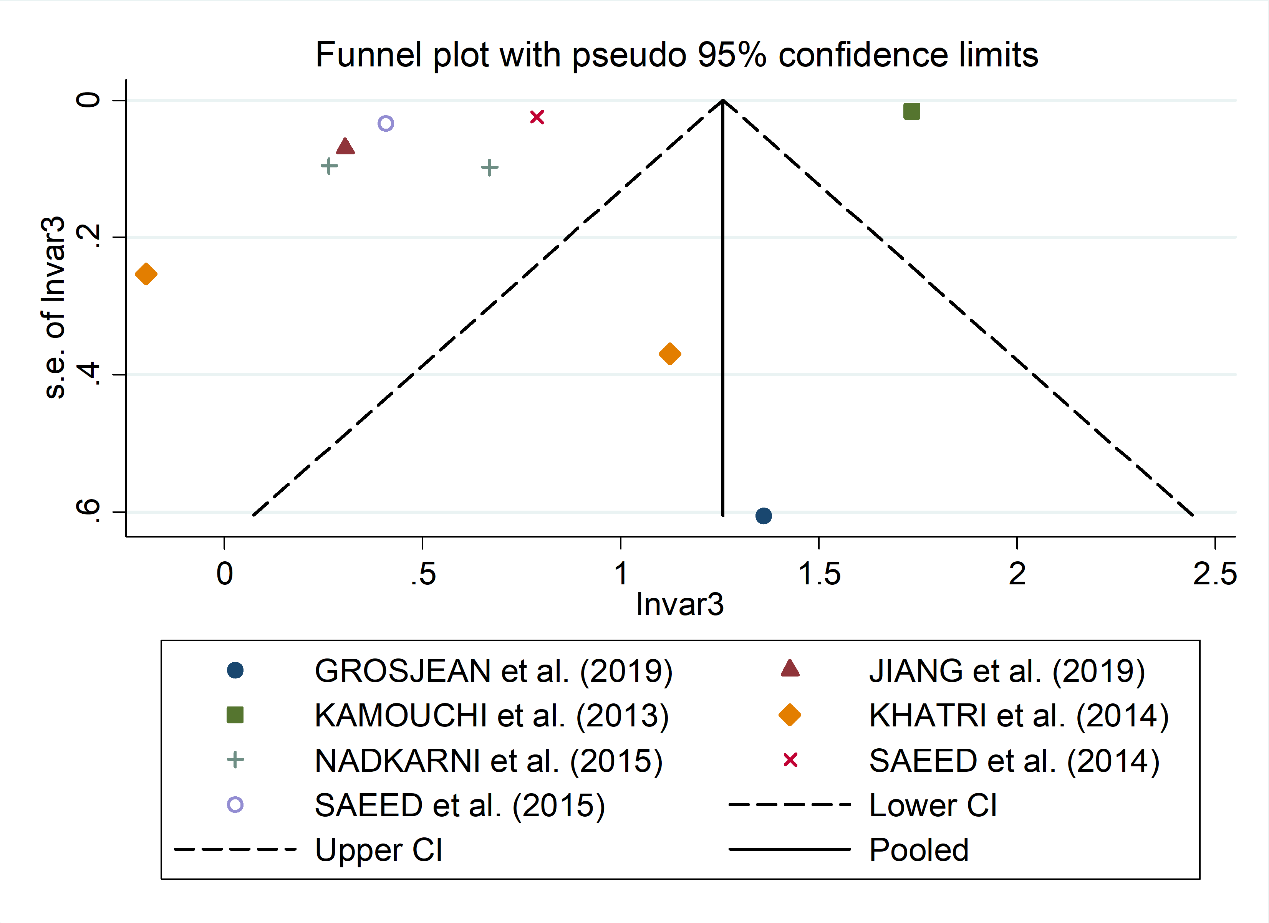


Supplementary figure 2. Funnel plots of studies exploring the association between AKI after stroke and in-hospital mortality. Abbreviation: AKI, acute kidney injury.


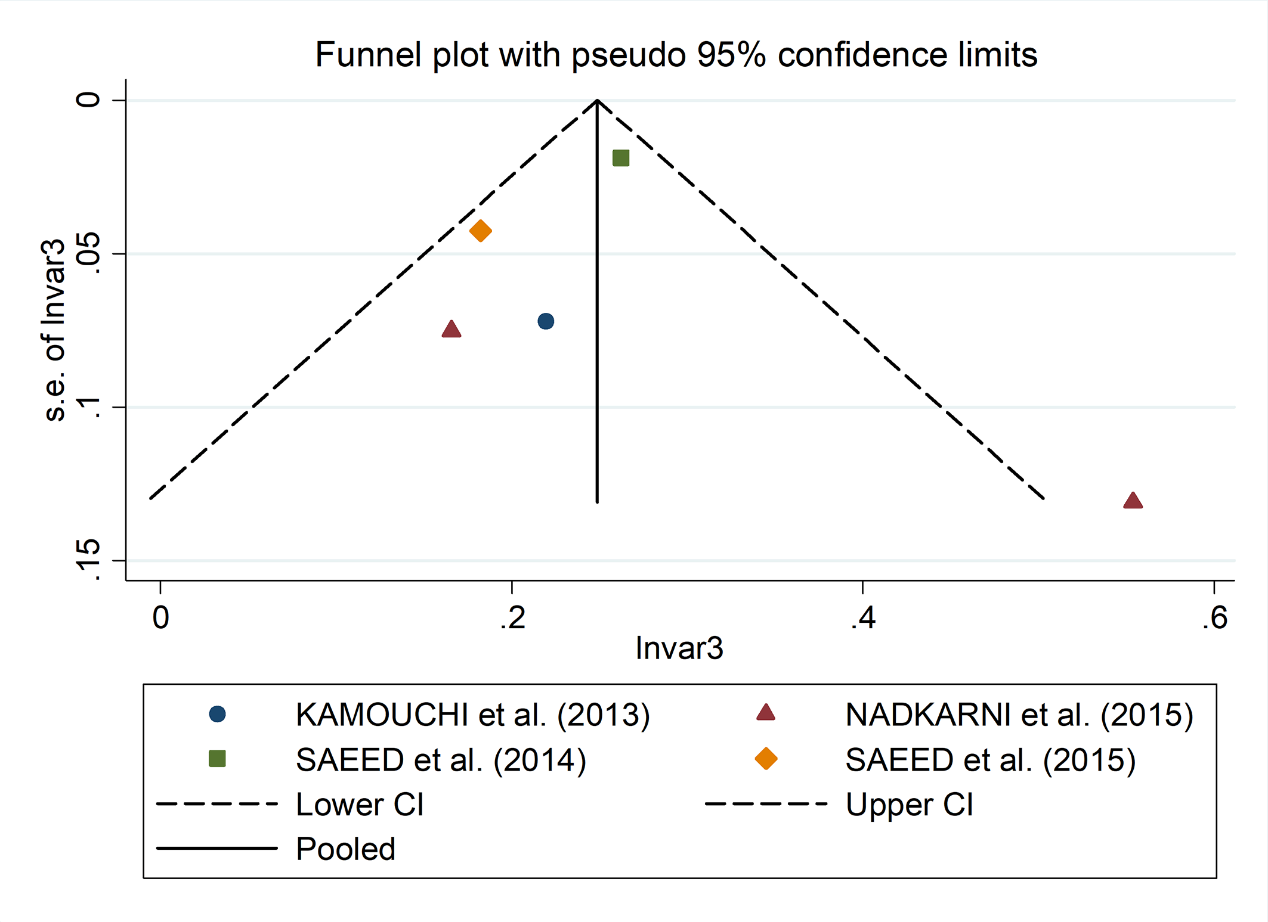


Supplementary figure 3. Funnel plots of studies exploring the associations between AKI after stroke and functional outcome. Abbreviations: AKI, acute kidney injury.

**Supplementary references**

1. Covic, A., Schiller, A., Mardare, N. G., Petrica, L., Petrica, M., Mihaescu, A., and Posta, N. (2008) The impact of acute kidney injury on short-term survival in an Eastern European population with stroke, Nephrology, dialysis, transplantation : official publication of the European Dialysis and Transplant Association - European Renal Association 23, 2228-2234.

2. Tsagalis, G., Akrivos, T., Alevizaki, M., Manios, E., Theodorakis, M., Laggouranis, A., and Vemmos, K. N. (2009) Long-term prognosis of acute kidney injury after first acute stroke, Clinical journal of the American Society of Nephrology : CJASN 4, 616-622.

3. Athilingam, P., King, K. B., Burgin, S. W., Ackerman, M., Cushman, L. A., and Chen, L. (2011) Montreal Cognitive Assessment and Mini-Mental Status Examination compared as cognitive screening tools in heart failure, Heart & lung : the journal of critical care 40, 521-529.

4. Kamouchi, M., Sakai, H., Kiyohara, Y., Minematsu, K., Hayashi, K., and Kitazono, T. (2013) Acute kidney injury and edaravone in acute ischemic stroke: the Fukuoka Stroke Registry, Journal of stroke and cerebrovascular diseases : the official journal of National Stroke Association 22, e470-476.

5. Saeed, F., Adil, M. M., Khursheed, F., Daimee, U. A., Branch, L. A., Jr., Vidal, G. A., and Qureshi, A. I. (2014) Acute renal failure is associated with higher death and disability in patients with acute ischemic stroke: analysis of nationwide inpatient sample, Stroke 45, 1478-1480.

6. Khatri, M., Himmelfarb, J., Adams, D., Becker, K., Longstreth, W. T., and Tirschwell, D. L. (2014) Acute kidney injury is associated with increased hospital mortality after stroke, Journal of stroke and cerebrovascular diseases : the official journal of National Stroke Association 23, 25-30.

7. Saeed, F., Adil, M. M., Piracha, B. H., and Qureshi, A. I. (2015) Acute renal failure worsens in-hospital outcomes in patients with intracerebral hemorrhage, Journal of stroke and cerebrovascular diseases : the official journal of National Stroke Association 24, 789-794.

8. Nadkarni, G. N., Patel, A. A., Konstantinidis, I., Mahajan, A., Agarwal, S. K., Kamat, S., Annapureddy, N., Benjo, A., and Thakar, C. V. (2015) Dialysis Requiring Acute Kidney Injury in Acute Cerebrovascular Accident Hospitalizations, Stroke 46, 3226-3231.

9. Mohamed, W., Bhattacharya, P., Shankar, L., Chaturvedi, S., and Madhavan, R. (2015) Which Comorbidities and Complications Predict Ischemic Stroke Recovery and Length of Stay?, The neurologist 20, 27-32.

10. Jiang, F., Su, L., Xiang, H., Zhang, X., Xu, D., Zhang, Z., and Peng, Z. (2019) Incidence, Risk factors, and Biomarkers Predicting Ischemic or Hemorrhagic Stroke Associated Acute Kidney Injury and Outcome: A Retrospective Study in a General Intensive Care Unit, Blood purification 47, 317-326.

11. Grosjean, F., Tonani, M., Maccarrone, R., Cerra, C., Spaltini, F., De Silvestri, A., Falaschi, F., Migliazza, S., Tinelli, C., Rampino, T., Di Sabatino, A., and Martignoni, A. (2019) Under-recognized post-stroke acute kidney injury: risk factors and relevance for stroke outcome of a frequent comorbidity, International urology and nephrology 51, 1597-1604.
